# The over-expression of a chrysanthemum gene encoding an RNA polymerase II CTD phosphatase-like 1 enzyme enhances tolerance to heat stress

**DOI:** 10.1038/s41438-018-0037-y

**Published:** 2018-07-01

**Authors:** Yuying Qi, Yanan Liu, Zixin Zhang, Jiaojiao Gao, Zhiyong Guan, Weimin Fang, Sumei Chen, Fadi Chen, Jiafu Jiang

**Affiliations:** College of Horticulture, Key Laboratory of Landscaping, Ministry of Agriculture, Nanjing Agricultural University, 210095 Nanjing, China

## Abstract

The enzyme RNAPII CTD phosphatase-like 1 is known as a transcriptional regulator of the plant response to various abiotic stresses. Here, the isolation of *CmCPL1*, a chrysanthemum (*Chrysanthemum morifolium*) gene encoding this enzyme is described. Its predicted 955 residue gene product includes the FCPH catalytic domain, two double-stranded RNA binding motifs, and a nuclear localization signal. A sub-cellular localization assay confirmed that CmCPL1 was expressed in the nucleus. *CmCPL1* transcription was shown to be significantly inducible by heat stress. The over-expression and knockdown of *CmCPL1*, respectively, increased and diminished the tolerance of chrysanthemum to heat stress, which maybe dependent on the regulation of CmCPL1 and on the expression of downstream heat stress-responsive genes.

## Crop genetics: plants that can take the heat

Researchers in China have engineered chrysanthemum plants with increased heat tolerance. Studies in model species have shown that the plant gene CPL1 is involved in their response to many kinds of stress, including temperature, drought, and salinity. Using the CPL1 sequence from studied species as a guide, Jiafu Jian’s team at Nanjing Agricultural University isolated the gene in chrysanthemum. They found that CPL1 is expressed most strongly in leaf cells and remains in the cells’ nucleus. CPL1 expression increased in plants which were exposed to stressful conditions, especially heat. When the researchers engineered chrysanthemum with artificially increased levels of CPL1, the plants were better able to tolerate heat; by contrast, plants with reduced CPL1 levels were more sensitive to heat stress. Altogether, these findings point the way towards breeding or engineering heat-tolerant chrysanthemum varieties.

## Introduction

RNA polymerase II (RNAP II) is a multi-subunit enzyme complex enabling the transcription of mRNA precursors, and microRNAs^1^. It is also involved in the regulation of various mRNA maturation processes, including capping, splicing, and polyadenylation^2,3^. The large RNAP II subunit's carboxyl terminal domain (CTD) includes a conserved heptapeptide (YSPTSPS), which plays important roles in the regulation of gene expression through its own phosphorylation/dephosphorylation^4^. The RNAPII CTD phosphatase FCP1 is largely responsible for the dephosphorylation of Ser-2 and Ser-5 present in the heptapeptide^5–8^. The RNAPII CTD phosphatase-like 1 enzyme (CPL1), a homolog of FCP1, harbors the FCPH domain and displays CTD phosphatase activity^9,10^. It also features two dsRNA-binding motifs (DSRMs) and a nuclear localization signal (NLS) at its C terminus, which are important for, respectively, the necessary protein–protein and protein–RNA interactions, and for nuclear localization^11–13^.

CPL1 proteins have been implicated, particularly, in *Arabidopsis thaliana*, in both the plant's development and its stress response^5,14–17^. According to Zhang et al.^6^, both the growth and morphogenesis of the plant are affected by the action of AtCPL1 on Rho GTPase signaling, while Xiong et al.^17^ have confirmed that the same enzyme contributes to the plant's response to low temperature, drought, and salinity stress by negatively regulating the genes encoding DRE/CRT and CBF/DREB proteins. The over-expression of *RCF2* (a *CPL1* allele) enhances the heat tolerance of *A. thaliana* through its control over the phosphorylation status of the transcription factor NAC019 and its influence over the activity of various heat stress transcription factors (HSFs) and heat shock proteins (HSPs)^18^. The loss-of-function of *AtCPL1* results in a transcriptional response similar to that induced by iron deficiency^19^. In addition, a number of interaction partners of CPL1 involved in the response to abiotic stress have been recently identified^20–22^. As yet, however, nothing is known regarding CPL1 function outside of the model plants.

Chrysanthemum (*Chrysanthemum morifolium*) is a leading ornamental species. The species is highly sensitive to a number of abiotic stresses, including drought, heat, salinity, heavy metal pollution, and nutrient deficiency^23^. Heat stress is known to be particularly damaging to both the growth and end-use quality of the chrysanthemum^24,25^. Supporting the breeding of improved cultivars would therefore benefit from a firmer understanding of the mechanistic basis of the chrysanthemum's adaptation to heat stress. Here, the goal was to characterize the *CmCPL1* gene and its product, and to explore the effect of its over-expression and knockdown on the plant's heat tolerance.

## Materials and methods

### Plant materials, growing conditions, and stress treatments

Cuttings of the chrysanthemum cultivar ‘Jinba’, conserved by the Chrysanthemum Germplasm Resource Preserving Centre (Nanjing Agricultural University, Nanjing, China), were cultivated in a 3:5 mixture of garden soil and vermiculite. The plants were transferred and pre-incubated in an artificial climate chamber delivering a 10-h photoperiod (300 μmol m^−2^ s^−1^ light), 70–80% relative humidity, and a day/night temperature regime of 28/22 °C. Plants at the 8–10 leaf stage were subjected to a variable period of exposure to a range of abiotic stresses, namely high (45 °C) and low (4 °C) temperature, salinity (200 mM NaCl), drought stress (20% PEG 6000), iron deficiency, and treatment with 100 μM abscisic acid (ABA). For the purpose of transcriptional analysis, the leaf material was sampled before the onset of stress (0 h), and then after 3, 6, 12, and 24 h, with the exception of the iron deficiency stress, where the first sampling was carried out at 0 h, and subsequent ones after 3, 5, 7, 9, and 11 days. The samples required for RNA extraction were snap-frozen in liquid nitrogen, then stored at −80 °C.

### Isolation of CmCPL1

Total RNA was extracted from the frozen leaf samples using the RNAiso Plus reagent (TaKaRa, Japan), following the manufacturer’s protocol. The resulting RNA was reverse-transcribed into the first cDNA strand using SuperScript III reverse transcriptase (Invitrogen, USA), following the manufacturer’s protocol. The primer pair CPL1-F/-R (sequences given in Table S1) was designed to amplify a fragment of *CmCPL1* based on the sequence represented in the transcriptome of cultivar 'Yuuka' (SRP029991), and RACE-PCR(rapid amplification of cDNA ends-Polymerase Chain Reaction); was then used to extend the sequence into the full-length cDNA (SMARTer^®^ RACE 5′/3′ Kit, Clontech). The resulting sequence, after its gel purification (Agarose Gel DNA Purification Kit, TaKaRa), was ligated into pMD19-T (TaKaRa) for sequencing. Finally, the CmCPL1-F/-R primer pair (Table S1) was designed to amplify the entire *CmCPL1* coding sequence (as confirmed by amplicon sequencing).

### Sub-cellular localization of CmCPL1 protein

The *CmCPL1* ORF was amplified using a forward primer (CmCPL1-SF) incorporating a *Sal*I restriction site and a reverse primer (CmCPL1-NR) with a *Not*I site (sequences given in Table S1). The resulting PCR product was transferred into pENTR1A (Invitrogen, USA) and subjected to an LR reaction (a recombination reaction between attL and attR sites); using the binary vector pMDC43^26^ in order to generate the transgene construct p*35S::GFP-CmCPL1*. The construct was transiently introduced into onion epidermal cells using a PDS-1000/He helium-driven particle accelerator (Bio-Rad, USA); control cells were transformed in the same way with p*35S::GFP*. After their transformation, the onion epidermis samples were held for 16 h at 25 °C in the dark, after which GFP (Green Fluorescent Protein) fluorescence was monitored by laser scanning confocal microscopy (Zeiss LSM780, Germany).

### Quantitative real-time PCR (qRT-PCR)

The first cDNA strand synthesized from the mRNA extracted from the leaf samples was used as the template for 20 µL qRT-PCRs formulated with SYBR^®^ Premix Ex Taq^TM^ II (TaKaRa) on a Mastercycler^®^ ep realplex Real-Time PCR System (Eppendorf, Germany). Each reaction was composed of 10 µL SYBR Green PCR master mix, 0.4 µL of each primer (10 µM), 4.2 µL H_2_O, and 5 µL of cDNA. The PCR cycling regime comprised an initial denaturation of 95 °C/30 s, followed by 40 cycles of 95 °C/5 s and 60 °C/30 s. Amplification of the *CmCPL1* sequence was driven by the primer pair qCmCPL1-F/R and that of the reference sequence *CmEF1α* (GenBank: AB679278.1) by the primer pair EF1α-F/R (sequences given in Table S1). Normalized transcript abundances were derived by applying the 2^−ΔΔCT^ method^27^.

### Chrysanthemum transformation

Artificial miRNAs (amiRNAs) designed to repress *CmCPL1* were prepared using a protocol slightly modified from the one described by Shida et al.^28^. The product amplified by the A and B primer pair (sequences given in Table S1) was inserted into pENTR1A via its *Sal* I and *Not* I sites, and from there into pMDC32 (Invitrogen, USA) using the LR reaction, finally generating the knockdown construct p*MDC32-CmCPL1_amiRNA*. The over-expression construct p*MDC32-CmCPL1* was obtained following the procedure given above for obtaining p*35S::GFP-CmCPL1*. The plasmids were introduced separately into *Agrobacterium tumefaciens* strain EHA105 using the freeze–thaw method, and from there into chrysanthemum following Mao et al.^29^. The abundance of *CmCPL1* transcript in the resulting transgenic and non-transgenic (NT) plants was detected using qRT-PCR analysis.

### Evaluation of heat tolerance

Rooted cuttings were grown to the 8–10 leaf stage, then exposed to a 24-h period of 45 °C; the plants were imaged after 0, 1, 3, 6, 12, 15, 20, and 24 h. Measurements were made of the leaves' maximal photochemical efficiency (Fv/Fm) after 0, 12, and 24 h, following the method given by Wang et al.^30^, and the free proline content and peroxidase (POD) activity of the leaf were assessed after 0, 1, 3, 6, 12, and 24 h, as described by Liu et al.^31^ and He et al.^32^, respectively. Each set of measurements was based on 25 plants per genotype (NT and transgenics), and the entire experiment was replicated three times.

### Transcription of heat-responsive genes

The abundance of transcript in both NT and transgenic plants generated from a number of heat stress-related genes (*HSP70*, *HSP*, *sHSP*, *HSFA2*, *DREB2A*, and *WRKY41*) was evaluated in triplicate using qRT-PCR. Leaf samples were collected after the plants had been exposed to 45 °C for 0, 1, 4, and 12 h. The *CmEF1α* sequence was used as the reference for normalization. The relevant primer sequences are given in Table S1.

### Statistical analysis

All analyses were performed using routines implemented within the SPSS v20.0 package (SPSS Inc., Chicago, IL, USA). A one-way analysis of variance, based on the Tukey test, was used to identify means differing significantly from one another; the significance thresholds were 0.05 (marked by *) and 0.01 (**).

## Results

### Isolation of CmCPL1 and the analysis of its nucleotide and peptide sequences

**Fig. 1:**
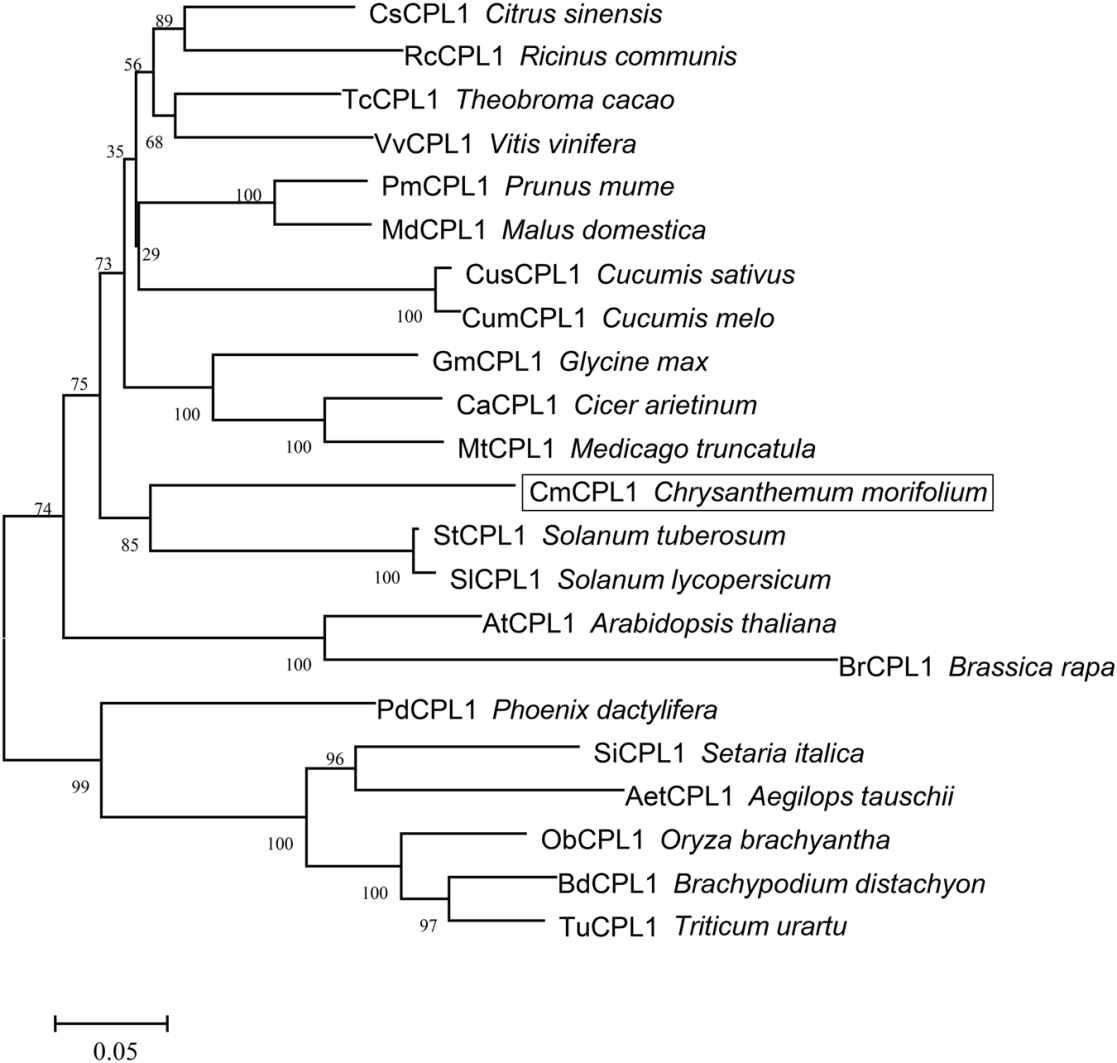
The phylogeny of plant CPL1s. The bootstrap values shown indicate the robustness of each branch. The scale bar represents 0.05 substitutions per site

The *CmCPL1* sequence isolated from cultivar 'Jinba' was 3159 bp in length, with 2868 bp of an open reading frame. The gene was predicted to encode a 955 residue protein (Fig. S1). A functional domain analysis of the predicted CmCPL1 protein showed that it featured a typical RNAPII CTD phosphatase catalytic domain (FCPH domain), two DSRMs, and one NLS (Fig. S1). A phylogenetic analysis demonstrated that the most similar sequences to CmCPL1 were StCPL1 from potato and SlCPL1 from tomato (Fig. 1).

### Sub-cellular localization of CmCPL1

**Fig. 2:**
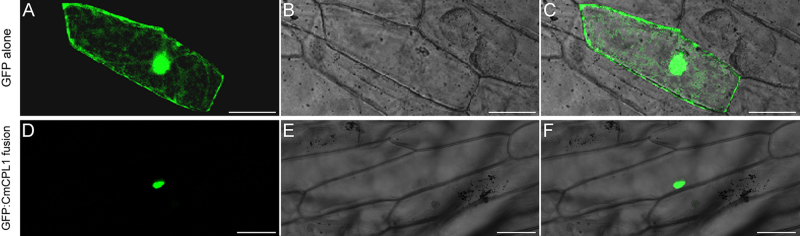
Sub-cellular localization of CmCPL1 in transiently transformed onion epidermal cells. Cells transformed with **a**–**c** p*35S::GFP*, **d**–**f** p*35S::GFP-CmCPL1*. **a**, **d** Dark-field images showing the GFP signal, **b**, **e** light-field images allowing the morphology of the cell to be visualized, **c**, **f** merged images of, respectively, **a**, **d** and **b**, **e**. Bar: 100 μm

The sub-cellular localization of CmCPL1 was inferred from the transient expression of *p35S::GFP-CmCPL1* in onion epidermal cells. The transformed cells expressed *GFP* strictly in the nucleus (Fig. 2d–f), whereas in control transgenic cells expressing p*35S::GFP*, GFP signal was detected throughout the cell (Fig. 2a–c).

### Transcription pattern of CmCPL1 in NT chrysanthemum

**Fig. 3:**
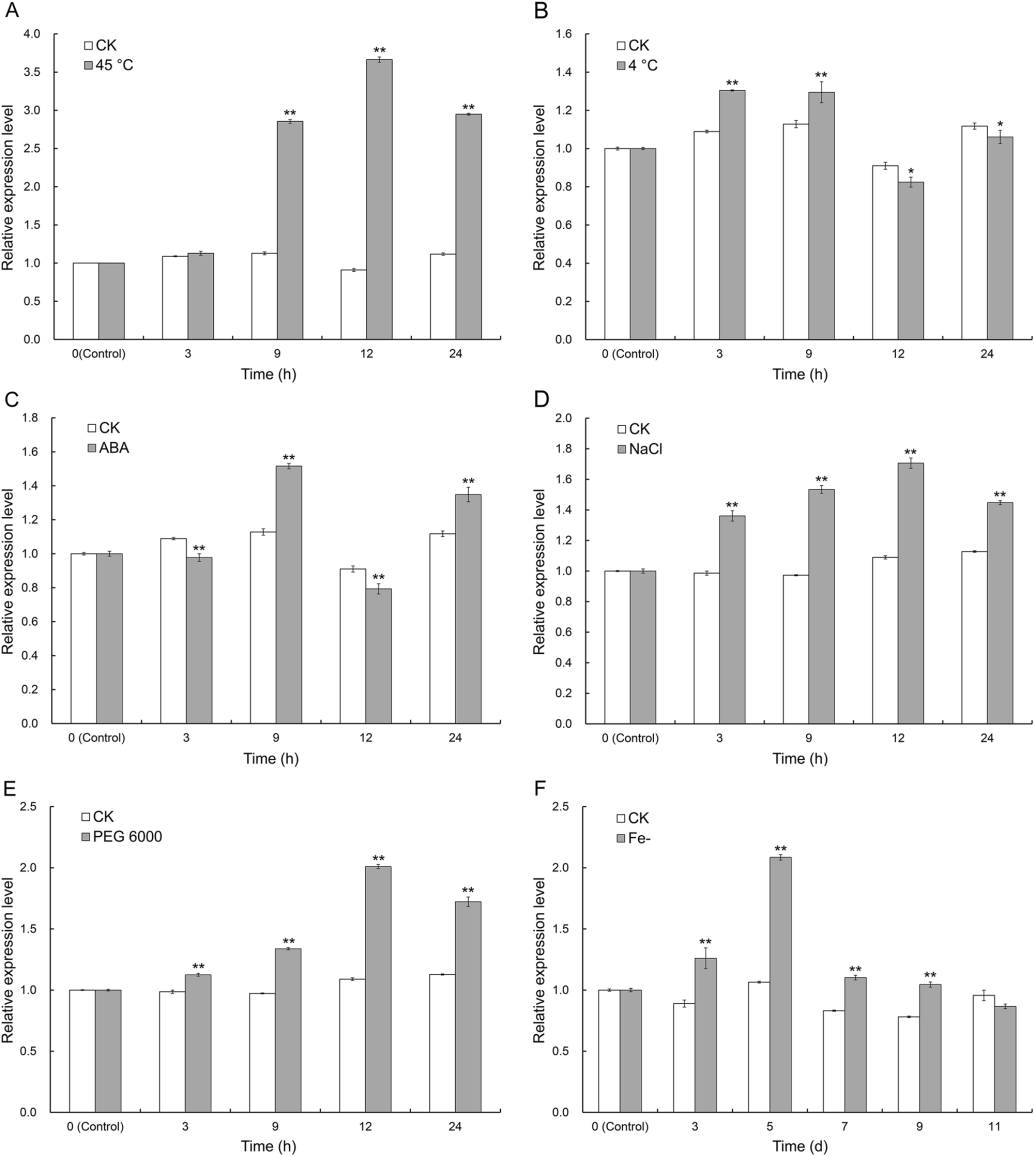
Transcription profiling of *CmCPL1* in plants exposed to abiotic stress. **a** Heat stress (40 °C), **b** low-temperature stress (4 °C), **c** treatment with 100 μM ABA, **d** salinity stress (200 mM NaCl), **e** drought stress (20% PEG 6000), **f** iron deficiency. Each value represents the mean of three biological replicates, and the whiskers indicate the SE. *, **: Treatment means differed from the control means (CK) at, respectively, *P* < 0.05 and <0.01

A qRT-PCR analysis showed that *CmCPL1* transcription was highest in the leaf, followed by the flower, root, and stem (Fig. S2). The gene was inducible by a range of abiotic stresses (Fig. 3); of particular note was its strong induction following the plants' exposure to 45 °C, which resulted in an approximately four-fold increase in transcript abundance over the untreated control after a 12-h exposure to stress (Fig. 3a).

### Obtainment of CmCPL1 over-expression and knockdown lines

**Fig. 4:**
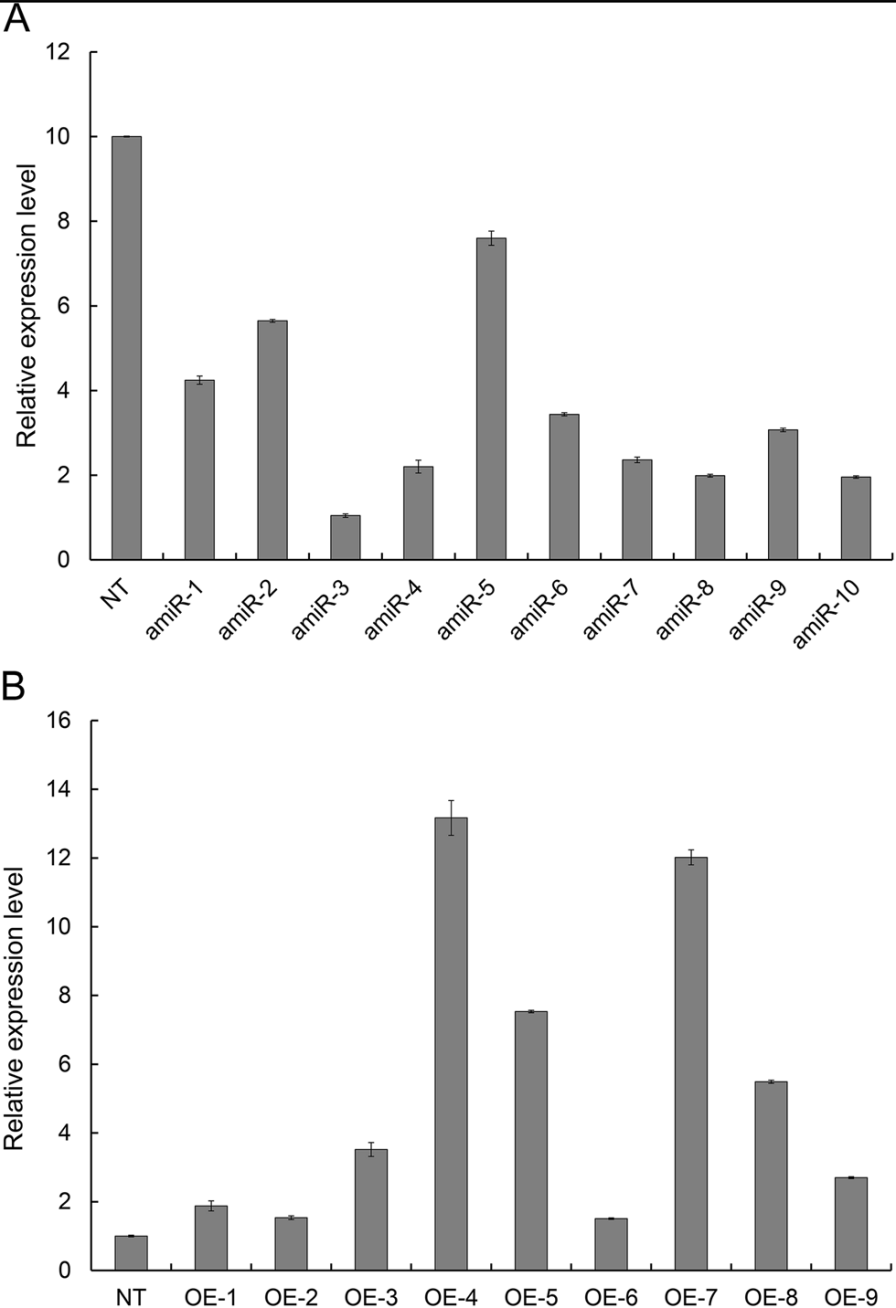
Abundance of *CmCPL1* transcript in transgenic and non-transgenic (NT) chrysanthemum plants. **a** *CmCPL1* knockdown plants and **b** *CmCPL1* over-expression plants

The over-expression of *CmCPL1* in cultivar 'Jinba' was achieved by introducing the transgene p*MDC32-CmCPL1* (Fig. S3A), while its knockdown was induced by the introduction of p*MDC32-CmCPL1_amiRNA* (Fig. S3B). Putative transgenic lines were regenerated on a selective medium (Fig. S4A-D) and validated using a genomic PCR assay (Fig. S4E). When the level of *CmCPL1* transcription in these plants was estimated using qRT-PCR, two of the p*MDC32-CmCPL1_amiRNA* plants (amiR-3 and amiR-8) showed clear evidence of knockdown (Fig. 4a), while the abundance of *CmCPL1* transcript was substantially higher in the p*MDC32-CmCPL1* lines OE-4 and OE-7 than in NT plants (Fig. 4b). Thus, these four transgenic lines were selected for evaluating the effect of *CmCPL1* transcript abundance on the plant's heat tolerance.

### The heat tolerance of transgenic chrysanthemum plants

**Fig. 5:**
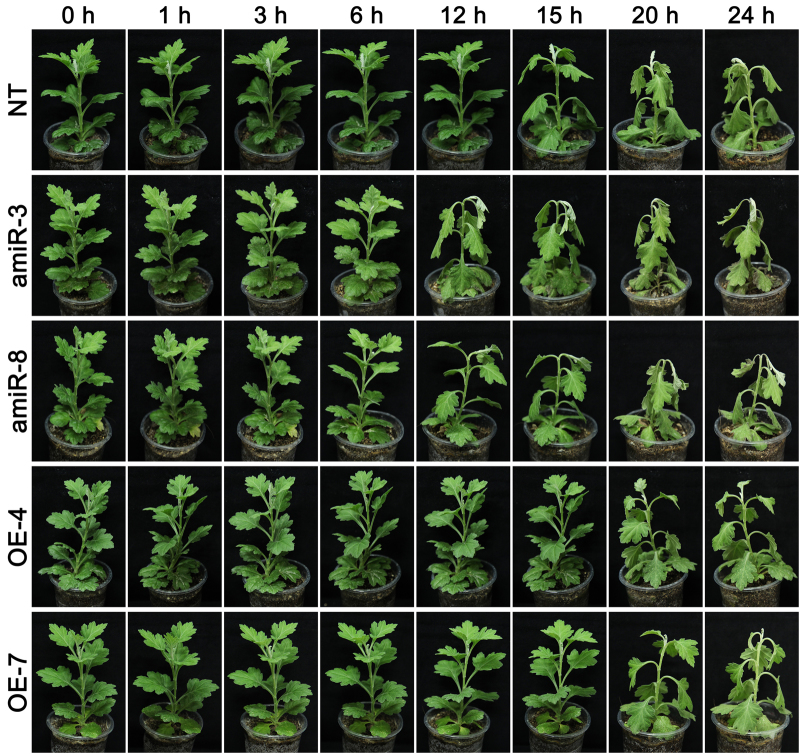
The phenotype of *CmCPL1* transgenic chrysanthemum plants exposed to heat stress. amiR-3, -8, *CmCPL1* knockdown plants; OE-4, -7, *CmCPL1* over-expression plants; NT, non-transgenic plants. The phenotype of transgenic (*CmCPL1* knockdown [amiR-3, -8] and over-expression [OE-4, -7]) and non-transgenic (NT) chrysanthemum plants exposed to heat stress

**Fig. 6:**
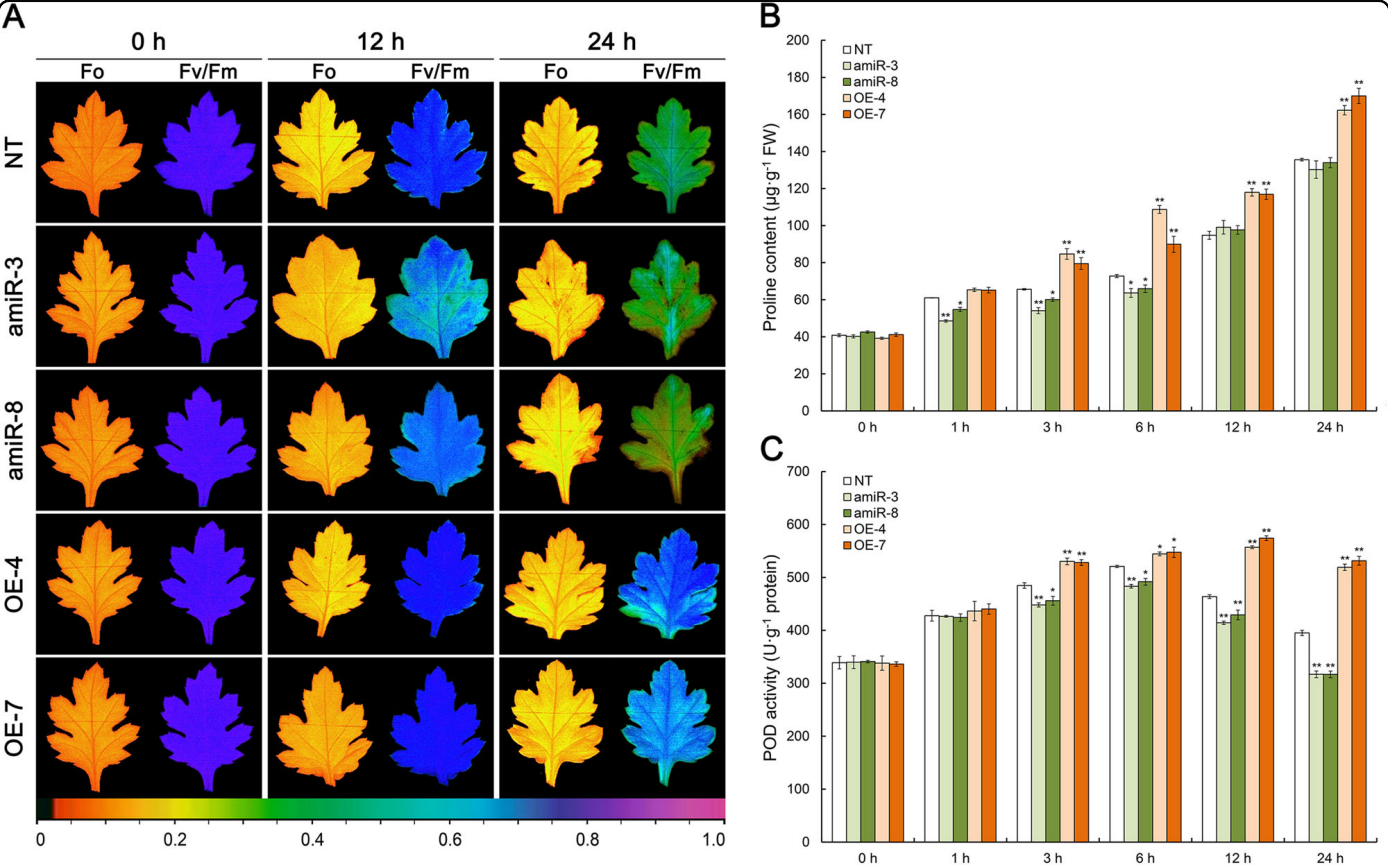
The effect of heat stress on physiological indicators in the leaves of *CmCPL1* transgenic chrysanthemum plants. amiR-3, -8, *CmCPL1* knockdown plants; OE-4, -7, *CmCPL1* over-expression plants; NT, non-transgenic plants. **a** Fv/Fm and Fo images at the end of the stress episode. The pseudocolored bar depicted at the bottom of the panel ranges from 0 (black) to 1.0 (purple). **b** The leaf content of free proline and **c** leaf POD activity. Values shown in the form of mean ± SE (*n* = 3). *, **: Treatment means differed from the control means (NT) at, respectively, *P* < 0.05 and <0.01

Heat stress induced leaf wilting in NT plants within 15 h, after which the severity of the wilting continued to rise; the two amiR line plants reacted more strongly than NT plants, and were severely wilted at the 24 h time point; however, leaves of the OE-4 and OE-7 line plants only began to wilt after a 20-h exposure (Fig. 5). There was little variation with respect to the photosynthesis parameter Fv/Fm between the four transgenic and the NT plants prior to the onset of stress; but as the stress period was prolonged, both amiR line plants recorded a lower Fv/Fm than NT plants, while both OE line plants recorded a higher one (Fig. 6a, S5). Similarly, while both proline content and POD activity were significantly enhanced by the stress in all genotypes, the levels were lower than NT plants in the amiR plants, and higher in the OE ones (Fig. 6b, c).

### Transcription regulation of several heat-responsive genes by CmCPL1

**Fig. 7:**
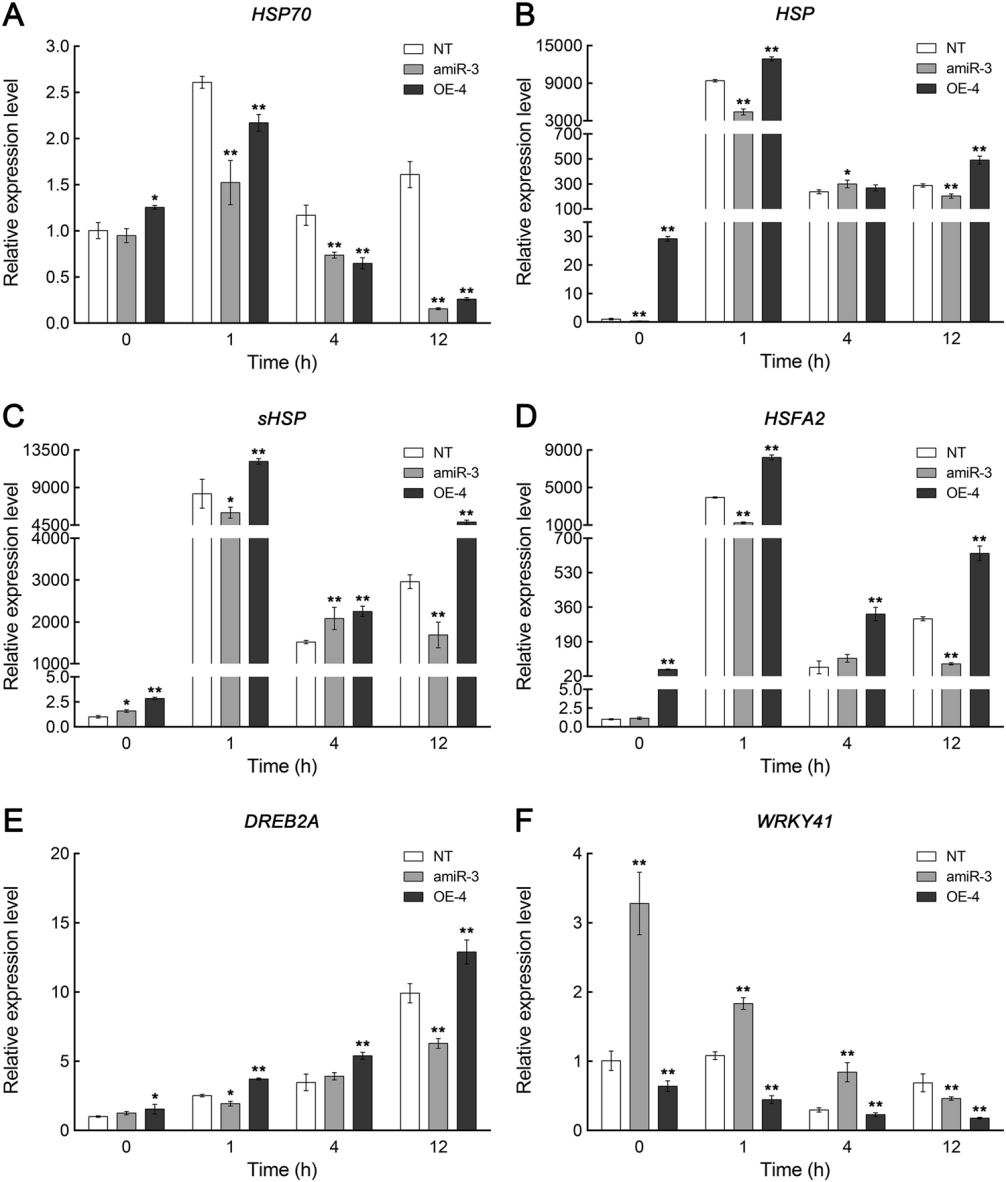
Abundance of transcript produced by various heat-responsive genes in transgenic (*CmCPL1* knockdown [amiR-3] and over-expression [OE-4]) and non-transgenic (NT) chrysanthemum plants exposed to heat stress. Values shown in the form of mean ± SE (*n* = 3). *, **: Treatment means differed from the control means (NT) at, respectively, *P* < 0.05 and <0.01

In both types of transgenic plants, the abundance of *HSP70* transcript was less than that in the NT plants during their exposure to heat stress (Fig. 7a). However, compared to NT plants, the level of transcription of *HSP*, *sHSP*, and *HSF* was significantly higher in OE-4 and significantly lower in amiR-3, especially after a 1-h exposure to stress (Fig. 7b–d). Similarly, the abundance of *DREB2A* transcript was lower in amiR-3 and higher in OE-4 (Fig. 7e). The transcriptional behavior of *WRKY41* was very different: the abundance of the transcript was significantly higher in the amiR-3 line than in the NT plants, and lower in OE-4. Overall, the conclusion was that the level of *CmCPL1* transcription positively affected that of *HSP*, *sHSP*, *HSFA2*, and *DREB2A*, and negatively that of *WRKY41*.

## Discussion

The phosphorylation/dephosphorylation of RNAPII CTD mediated by various phosphatases is an important component of eukaryotic transcriptional regulation and mRNA processing^3^. The CPL1 protein, which acts to dephosphorylate the RNAPII CTD, is known to regulate not just the growth and development of plants, but also their stress responses^33^. Here, the product of a chrysanthemum gene (*CmCPL1*) encoding an RNAPII CPL1 protein has been shown to affect the plant's' response to heat stress. The CmCPL1 protein included the characteristic RNAPII CTD phosphatase catalytic domain, along with two DSRMs, mirroring the structure of AtCPL1^14,17^. Previous studies have demonstrated that CPL1 proteins are deposited in the plant cell nucleus, thanks to their C terminal NLS^5,18^. The CmCPL1 sequence similarly included a putative C terminal NLS, matching that carried by its homologs in both *A. thaliana* and rice^5^. Thus, as predicted, when onion epidermal cells were transiently transformed with the p*35S::GFP-CmCPL1* construct, GFP accumulated preferentially in the nuclei, confirming CmCPL1 to be a nuclear protein, presumably thanks to its C terminal NLS.

CPL1 is a key player in the plant's response to various abiotic stresses^5,14–17^. Since the absence of AtCPL1 results in heightened iron deficiency signaling and enhanced crosstalk with a branch of the osmotic stress/ABA signaling pathway, it has been proposed that this protein acts as a negative regulator of the above process^19,22^. Similarly, AtCPL1 has been described as a negative regulator of stress-responsive gene transcription in *A. thaliana* plants subjected to a range of abiotic stresses^14^. According to Guan et al.^18^, the allele of AtCPL1 (RCF2) controls the low temperature-responsive genes in both positive and negative manners, and is a major positive regulator of heat stress-responsive gene expression and themotolerance in *A. thaliana*. Here, the abundance of *CmCPL1* transcript was altered by the imposition of a range of abiotic stresses, which indicates that its product is an element of the general stress response of chrysanthemum. Of particular note was the heightened responsiveness of *CmCPL1* transcription to heat stress, suggesting the centrality of this protein's involvement in the heat stress response.

In the chrysanthemum plants subjected to heat stress, those which had beeen engineered to over-express *CmCPL1* displayed an enhanced level of tolerance, while the *CmCPL1* knockdown plants proved to be more sensitive than NT plants. A corresponding experiment in *A. thaliana* produced a completely similar result^18^. The expression of *CmCPL1* likely contributed to the physiological homeostasis of heat-stressed plants, specifically with respect to their photosynthetic efficiency, osmotic pressure of the cell, and the production of POD. Unfortunately, the contribution of *CmCPL1* to stress tolerance cannot exist extensively in other abiotic stress, such as salinity stress, indicating that CmCPl1 may have a specific role in thermotolerance (Fig. 6S). Recently, the genes encoding certain chrysanthemum HSPs and transcription factors have been shown to be inducible by heat stress^23,34^. In heat-stressed *A. thaliana* plants, the absence of AtCPL1 suppresses the transcription of a number of *HSFs*, *HSPs*, and *DREB2*, while the stress has the opposite effect on over-expressors of *AtCPL1*^18^. Here, the observation was that the presence of CmCPL1 was associated in heat-stressed plants with the upregulation of *HSP*, *sHSP*, *HSFA2*, and *DREB2A*, and the downregulation of *WRKY41*, in accordance with the outcome of similar experiments performed in other species^18,34^. Interestingly, the expression of *HSP70* was inhibited both in over-expression and knockdown plants under heat stress, implying that CmCPL1 was involved in the regulation of *HSP70* transcription, although this process may be complex and multi-factoring, which requires further study. The overall implication was that CmCPL1 acts as a positive regulator of a suite of heat stress-responsive genes, the products of which are required to protect the plant from damage imposed by heat stress. The gene therefore represents an interesting target for devising a genetic engineering-based strategy aimed to develop chrysanthemum cultivars expressing heightened heat tolerance.

## Electronic supplementary material

Table S1

Figure S1

Fiigure S2

Fiigure S3

Fiigure S4

Fiigure S5

Fiigure S6
