# Supplementary material for: The over-expression of a chrysanthemum gene encoding an RNA polymerase II CTD phosphatase-like 1 enzyme enhances tolerance to heat stress
Source: Hortic Res. 2018 Jul 1;5:37. doi: 10.1038/s41438-018-0037-y (PMC6026497; doi:10.1038/s41438-018-0037-y)
Supplement: Supplementary file 1 — Table S1 [file 41438_2018_37_MOESM1_ESM.docx]

**Supplementary materials**

**Table S1.** Primer names and sequences used in this study

| Primer Name | Sequence (5’ to 3’) | Annotation |
| --- | --- | --- |
| CPL1-F | TGTAAATGGGAGCAAAGAA | Fragment of CmCPL1 |
| CPL1-R | TGAAATGTCATGCCTAAACC |  |
| CmCPL1-F | ATGTATAAATCATCAAAGGT | Full-length of CmCPL1 |
| CmCPL1-R | TCAAGGAACAGGAGAGGCA |  |
| EF1α-F | TTTTGGTATCTGGTCCTGGAG | qRT-PCR for *CmEF1α* |
| EF1α-R | CCATTCAAGCGACAGACTCA |  |
| qCmCPL1-F | GCTGATAAGTATTTAGCTCGTCC | qRT-PCR for *CmCPL1* |
| qCmCPL1-R | TAGGTCCTCTTCTGGAAACATCG |  |
| CmCPL1-SF | ACGCGTCGACATGTATAAATCATCAAAGGT | For over-expression vector pMDC32-CmCPL1 |
| CmCPL1-NR | ATAAGAATGCGGCCGCTATCAAGGAACAGGAGAGGCA |  |
| I miR-s | gaTCATGTTAACCCGACTCCCTTtctctcttttgtattcc | For artificial microRNA expression vector pMDC32-CmCPL1 amiRNA |
| II miR-a | gaAAGGGAGTCGGGTTAACATGAtcaaagagaatcaatga |  |
| III miR*s | gaAAAGGAGTCGGGTAAACATGTtcacaggtcgtgatatg |  |
| V miR*a | gaACATGTTTACCCGACTCCTTTtctacatatatattcct |  |
| A | CTGCAAGGCGATTAAGTTGGGTAAC |  |
| B | GCGGATAACAATTTCACACAGGAAACAG |  |
| Hyg-F | CTTCTACACAGCCATCGGTCCAG | For genomic PCR detection |
| Hyg-R | CGGAAGTGCTTGACATTGGGGAG |  |
| HSP70-F | GCTTGCTGAGGCGGATGAGT | qRT-PCR for *HSP70*^1^ |
| HSP70-R | ACCTGATGGTGCGGGTTCCTC |  |
| HSP-F | GCATGGGAAGCATTGACATA | qRT-PCR for *HSP*^2^ |
| HSP-R | AAGGAAAAATGACCTTGCCA |  |
| sHSP-F | ATGCGAAAATGGAGGAAGTG | qRT-PCR for s*HSP*^2^ |
| sHSP-R | AGCCTGCAATGTCAATAGCC |  |
| HSFA2-F | CAAAGGAGGGTTTGAACGAA | qRT-PCR for *HSFA2*^2^ |
| HSFA2-R | CTTGTCGGATCCTCAACCAT |  |
| DREB2A-F | GGCAGCTGGTTATCCTATGC | qRT-PCR for *DREB2*^2^ |
| DREB2A-R | CCCCATCCAAGAAGTCAAAA |  |
| WRKY41-F | GCCAAATGACTTTCCAGCAT | qRT-PCR for *WRKY*^2^ |
| WRKY41-R | TCAGATCGTACGCTTTGTGG |  |

^1^Song A, Zhu X, Chen F, Gao H, Jiang J, Chen S (2014) A chrysanthemum heat shock protein confers tolerance to abiotic stress. *International Journal of Molecular Sciences*, 15: 5063-5078.

^2^Sun J, Ren L, Cheng Y, Gao J, Dong B, Chen S, Chen F, Jiang J (2014) Identification of differentially expressed genes in *Chrysanthemum nankingense* (Asteraceae) under heat stress by RNA Seq. *Gene*, 552: 59-66.

**>HSP70 (GenBank: AB503697**^1^**)**

ATGGCTGGTAAAGGTGAAGGACCTGCTATTGGTATTGATCTCGGGACCACTTATTCATGTGTTGGTGTATGGCAACACGACCGTGTTGAAATTATCGCTAATGATCAGGGTAACAGAACGACGCCGTCTTATGTTGCCTTTACTGATCCTGAGAGGCTTATTGGTGATGCCGCCAAGAATCAGGTTGCCATGAACCCTACCAACACTGTTTTCGATGCTAAACGTCTCATCGGTAGGCGATTTTCTGACGCTTCAGTCCAAAGTGACATCAAGTTGTGGCCATTCAAGGTTACCCCTGGACCAGCTGAGAAGCCTATGATCGCTGTTAACTACAAGGGTGAGGAGAAGACCTTTGCCGCTGAGGAAATCTCTTCTATGGTTCTTATCAAGATGAAGGAAATCGCTGAGGCTTTTCTTGGTTCAACCGTTAAGAACGCTGTCGTTACAGTCCCAGCCTACTTCAACGACTCACAACGTCAAGCCACCAAGGATGCCGGAGTCATCTCTGGTCTCAATGTTATGCGTATTATTAACGAGCCAACTGCTGCTGCCATCGCTTACGGTCTTGACAAGAAGGCTACCAGCGTTGGTGAGAAGAATGTGCTCATCTTTGATCTTGGTGGTGGTACCTTCGATGTGTCACTTTTGACCATTGAAGAAGGTATCTTTGAAGTTAAAGCCACAGCCGGAGACACCCATCTTGGTGGTGAAGATTTTGACAACAGAATGGTTAACCACTTTGTTCAAGAGTTCAAGAGGAAGCACAAGAAGGACATCACTGGTAACCCCAGAGCCCTTAGAAGGTTGAGGACATCTTGTGAAAGAGCAAAGAGAACCCTCTCATCAACTGCTCAAACCACCATTGAAATTGACTCTCTATACGAAGGTGTTGACTTTTACTCCACCATCACCCGTGCCAGATTTGAGGAATTGAACATGGACTTGTTCAGAAAGTGTATGGAGCCAGTTGAGAAGTGTTTGAGAGACGCCAAGATGGACAAGAGCTCAATCCATGATGTTGTTCTTGTTGGTGGATCCACCCGTATCCCCAAGGTTCAGCAGCTTCTTCAGGATTTTTTCAATGGAAAGGAGCTCTGCAAGAGCATTAACCCAGATGAGGCTGTTGCCTACGGTGCTGCTGTCCAAGCCGCTATCTTGAGTGGTGAGGGCAATGAGAAGGTCCAGGACTTGCTTCTTTTGGATGTTACCCCATTGTCTCTTGGTCTTGAGACTGCTGGAGGTGTTATGACTGTTCTTATCCCAAGAAACACAACCATCCCAACCAAGAAGGAGCAAGTCTTCTCCACCTACTCTGACAACCAGCCTGGTGTGTTGATCCAAGTGTATGAAGGTGAAAGAACTAGAACCCGTGACAACAACTTGTTGGGCAAGTTTGAGCTTTCTGGAATTCCACCTGCTCCTCGTGGTGTCCCACAGATCACAGTCTGCTTTGACATCGATGCCAATGGTATCCTTAATGTCTCAGCTGAAGACAAGACCACCGGACAGAAGAACAAGATTACCATCACCAATGACAAGGGTAGACTCTCAAAGGAGGAAATTGAGAAGATGGTTCAGGAGGCTGAGAAATACAAGTCTGAGGATGAGGAGCACAAGAAGAAGGTTGAGGCCAAGAATGCTCTTGAGAACTACGCCTACAACATGAGGAACACTGTGAAGGATGAGAAGATCGGTGAGAAGTTGACCCCTGGTGACAAGAAAAAGATTGAGGATGCTATCGATGAGGCCATTGCATGGTTGGACAGCAACCAGCTTGCTGAGGCGGATGAGTTTGAAGACAAGATGAAGGAGCTAGAGAACGTGTGCAACCCGATCATTGCTAAGATGTACCAGGGAGGAGCTGGTGATGCTGCAGGAGGCATGGATGAGGAACCCGCACCATCAGGTGGCGGTGCTGGTCCCAAGATCGAAGAGGTCGACTAA

**>HSP (Unigene47971, SRA: SRP032828**^2^**)**

ATGAGCATGGGAAGCATTGACATAGGTGGTCTAGTGAGCCATTTGCTAGATGTTCCTGATACAATTGGCAAGGTCATTTTTCCTTCTAATGGGACACATCACGAGTCGCGAAATGTCGAATCGAAAGGCGGAAACATTCCTGTCGATATTGTTGACACTCCTAAGGAATATGTTTTGTACATGGATGTCCCTGGGTTGTCCAAGTCCGATATTCAGGTGACAGTGGAAGAGGAAAACTTATTGGTGGTGAAAAGCAATGGCAAGAGGAAGCGTGACGAAAAGGATGAGGAGGAGGAAGAAGGGTGCAAGTACTTGCGGCTCGAGAGGAGGCCAAAGAAGAACCTCATGAGGAAATTCCGTCTGCCCGAAAACTGTAACATGTCTGCTATCAGTGCAAAGTGCGAAAACGGGGTGTTGAAGGTGGTGGTTGAGAAGCTCCCTCCTCCGCCTAAGTCCAAGACTGTCCAAGTTGCGGTTTCGTAA

**>sHSP (Unigene1043, SRA: SRP032828**^2^**)**

CCATTCCAAGGCTTCCCTAGTGCACTTACCAACCTCCCTGACTCCTCTAGAGAAACCGCAGCAATTGCGAACACAAGGATCGACTGGAAGGAGACACCTGAGGCTCACGTCTTCAAGGCGGACTTACCCGGGCTTAAAAAGGAAGAAGTTAAGGTTGAAGTTGAGGAAGGGAGGGTGTTGCAGATAAGTGGTGAGAGAAGCAAGGAGAATGAAGAGAAGAATGATAAGTGGCATAGAGTTGAGAGAAGCTCAGGTAAGTTTATGAGGAGGTTTAGGTTGCCGGAGAATGCGAAAATGGAGGAAGTGAAGGCGTCCATGGAGAACGGTGTGCTAACTGTGACCGTGCCGAAAATGGAAGAGAAGAAGAAGGAGGTGAAGGCTATTGACATTGCAGGCTGA

**>HSFA2 (Unigene37791, SRA: SRP032828**^2^**)**

ATGCAGACAATGAAGGAAGAAGAAGTGACCTTCATCTCATCATCATCCTCATCATCCTCGTCATCACCAAGGCCAAAGGAGGGTTTGAACGAAGTGGGCCCACCTCCATTTCTGAGAAAGATATTCGACATGGTTGAGGATCCGACAAGTGACGAGGTGGTGTCGTGGAGCAAGACGAGGAATAGTTTTGTTGTGTGGGATTCGTATCGGTTTTCGACTGCTTTGTTGCCTAAGTATTTTAAGCATAGTAATTTTTCCAGCTTTGTAAGGCAGCTCAATACTTATGGTTTCAAGAAAGTGGATCCAGACAGATGGGAATTTGCGAATGAAGGGTTTCTGGGAGGTCAAAGACATTTGCTCAAAACAATTAGAAGAAGACGAAACGTGGCACAATCTGTCCAACCGAAGCAAGAATATGATCCATGTATCGAAGTAGGCCAATATGGGATTGAGGAAGAGCTTGAAGGACTAAAACGAGAAAGAAGCGTGTTAATGGCGGAGATAGTAAGACTGAGGCAACTACAACAACATTCACAAGACCAACTCATATCAATCGAAAACCGATTAAAAGCCACAGATCGAAAACAACAAAACATGATGGGATTCCTTGCAAAAGCGTTTAGCAACCCCAAGTTCCTACAGAAATACATTGATAAGAATGCAAACAAGGAGCAAAAACATATAGAAATTGGGCGTAAGAGGCGTTTAACAATGACGCCAAGTGTTGAGAATTTAACTGAGAATATCGACCCGTTCATTACAGCCCCGTTTGAAGAGGAATCAAGCAGCAATGTTGGTTTAGTGGAAGATGAGTCTCATGATAATTTATGGGAGGAACTGCTGAATGTCGATCTTCCGACTGAGAAAGCACCCGATGATTTTCTTGACGATTTAGATGGTCAGCAGCTTGATTGGGACGAGGATGTGCAAGAATTAGTGGATCAAATGGAATATCTAAGGTCATCCAATGCTTGA

**>DREB2A (Unigene26932, SRA: SRP032828**^2^**)**

ATGATTTCTGTTGGCTTTATTTTGGATACTAACCATTCTGTTTTCTTTGTTTACAGAATGACTGAAATGACTCTTCTTAGTCAACCTTCCAATACAGCTTGTGTTCCTAAGGGTAACTCAAAGAAGAGGACTGCTTCTAAATCAATGGATAAATTAACGAAAAGGCAAAAGGGAAATAGGGCTGAGGCACCAAAGATCGTGGCTGAGAGACTCGCCAAGTGGATTGAATATAACAAAGATGGAAAAGCAATAACGCGTAAAGCTCCTGCTAAAGGATCAAAAAAAGGCTGCATGAAAGGTAAAGGTGGGCCAGAAAATGGCAGGTGTAGTTTCAGAGGCGTGAGGCAAAGAACGTGGGGTAAATGGGTAGCTGAAATACGTGAACCAAATAAAGGTAAGAGGTTGTGGCTTGGGACTTTCGCCTCTGCTGTTGAAGCTGCTCTGGCATATGATAAAGCTGCTAGAGCAATGTATGGCGAGTATGCACGGATCAACTTGCCAAACTATCACACGAATACACTGTTGCCGTGTAATGCTGCTACTAGTTGCAACTCAGCTACTACCTGTAGTAACTCTGAGTGCAGTGAAGTTCAGGATTCAAAGTCCGGGTTAGACTGGTGTTCACCTACAGAGACTGTATGTGAAGCAAGTGAAGTCGTTAATGAATCACTGGATATTAAAGATGCTGCGGGTGTTTCTAATGATGATGGTTGCTATCAGGGATTTTCTGAGGATGATATGTTTTTCATAGACGAGCTTCTCGGTGGTACAGATCAGGAAAGTGAATTTAACAACGAGGCAGCTGGTTATCCTATGCATGATAATTCATGGTATGAGAACAAACAAGATGTCAGTCAAAATGAGTCCATATATATGCAGCAAGATGCTTGTGGGGTTGCACCGGACTTTGGTTTTGACTTCTTGGATGGGGGGCTTGAAGAATGTAACTTCTCACTCGAGGAGTTTGGTCTGTCCTTGGATCCTACTGAATCAGTTATGTAG

**>WRKY41 (Unigene10297, SRA: SRP032828**^2^**)**

ATGGAAAGTGCTTGTGTTTATGGACAGAAGACACTCATCAATGAACTAAGTCAAGGGTTACAAATGGCTAAACAGCTAAAGGGTAATCTTAATTCACCTGAAGCAAGAGATATCATACAGAAGATATTGGCTTCATATGACAACGCGTTGTTAGTTCTCAAATCAGGCGAGTCTGCTGGGCAGCGTGAGCCAAATGACTTTCCAGCATCCAGTCTGACCGAGTCATTGATAACCATAGCGAGCCCACAAAGCGTACGATCTGAGTTTAATCAGCCTTTTTCCAATGAGCACGACCCAAATGTTGTTTCCAAAAAGAGAAAGGGTTCGACAGTATGTGAAGATCAAGTTAAAATGTGCACTGATGATGGGTTAGAGGGTAGCGTTGATGATGGTCACAGTTGGAGAAAGTATGGACAGAAAGACATTTTGGGTGCCAAATTTCCAAGGAGCTATTATAGATGCACATACCGCAAGGCTGAGAAGTGCTTGGCGACAAAACAAGTACAGAGAACCGATGCAAATCCCACAGTATTTGATATCACGTACAAAGGAAAACACACTTGCAACCATCACGCTCGTTTGGCCGAACCACCTTTGCCTGAAAAACATGAAATAAACACATCCCACCACCAACAACTATCACGACCGAATCCGGGTGAAATGCTTTCAAATCTGAGAGCCAACCTCACTGTTAACACCTCGGATTTTGGTGCTACTGATCCACACTCGTTTTCCTTCCCTTTAGAACCATTTGGCATTATTGAAGATTACCAACAACTTCATTTGCCTAATGATTTTGATGATGAATTGTTGCAAGTCTATTCGCCACCTTTTATCTCCCCGGGCACTTCTGATTCAAACTGCTACACGGACTGGGACAGTTCACCATCACTAAATTTCACGGCAGATTTAGACCGTGATTTCAAATTTTAA
