# Supplementary figures and images for: The over-expression of a chrysanthemum gene encoding an RNA polymerase II CTD phosphatase-like 1 enzyme enhances tolerance to heat stress
Source: Hortic Res. 2018 Jul 1;5:37. doi: 10.1038/s41438-018-0037-y (PMC6026497; doi:10.1038/s41438-018-0037-y)

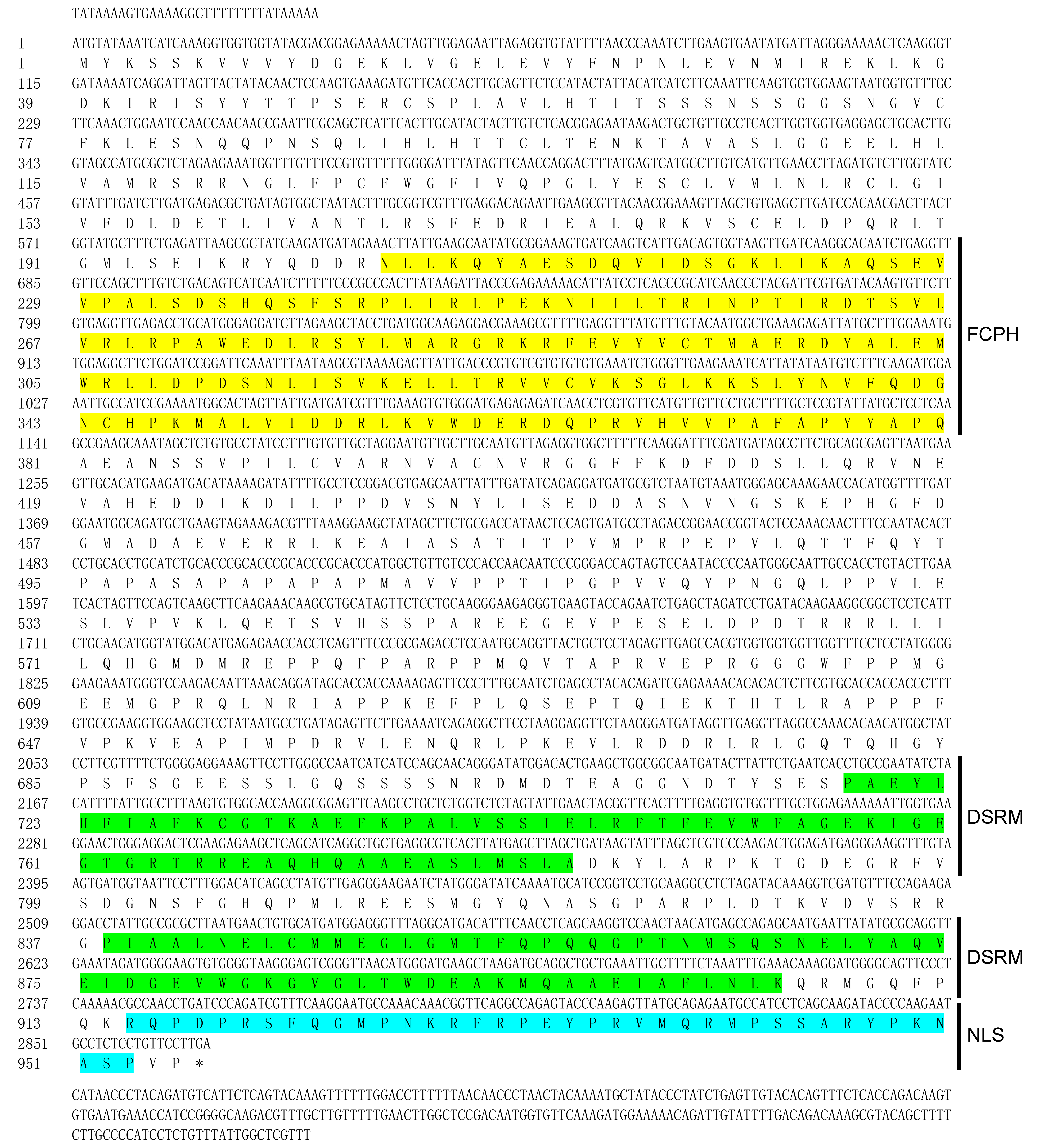

Supplement: Supplementary file 2 — Figure S1 [file 41438_2018_37_MOESM2_ESM.tif]

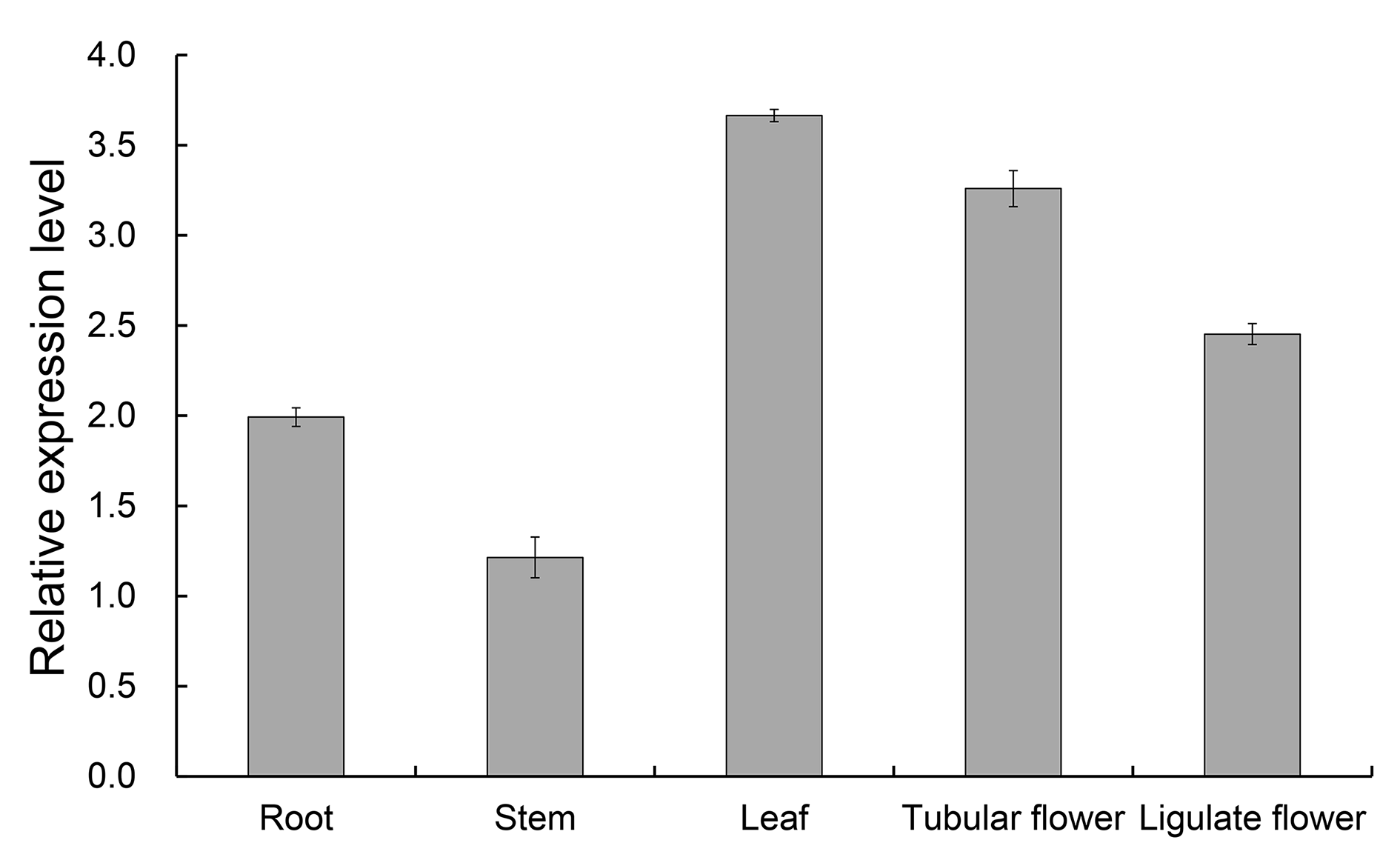

Supplement: Supplementary file 3 — Fiigure S2 [file 41438_2018_37_MOESM3_ESM.tif]

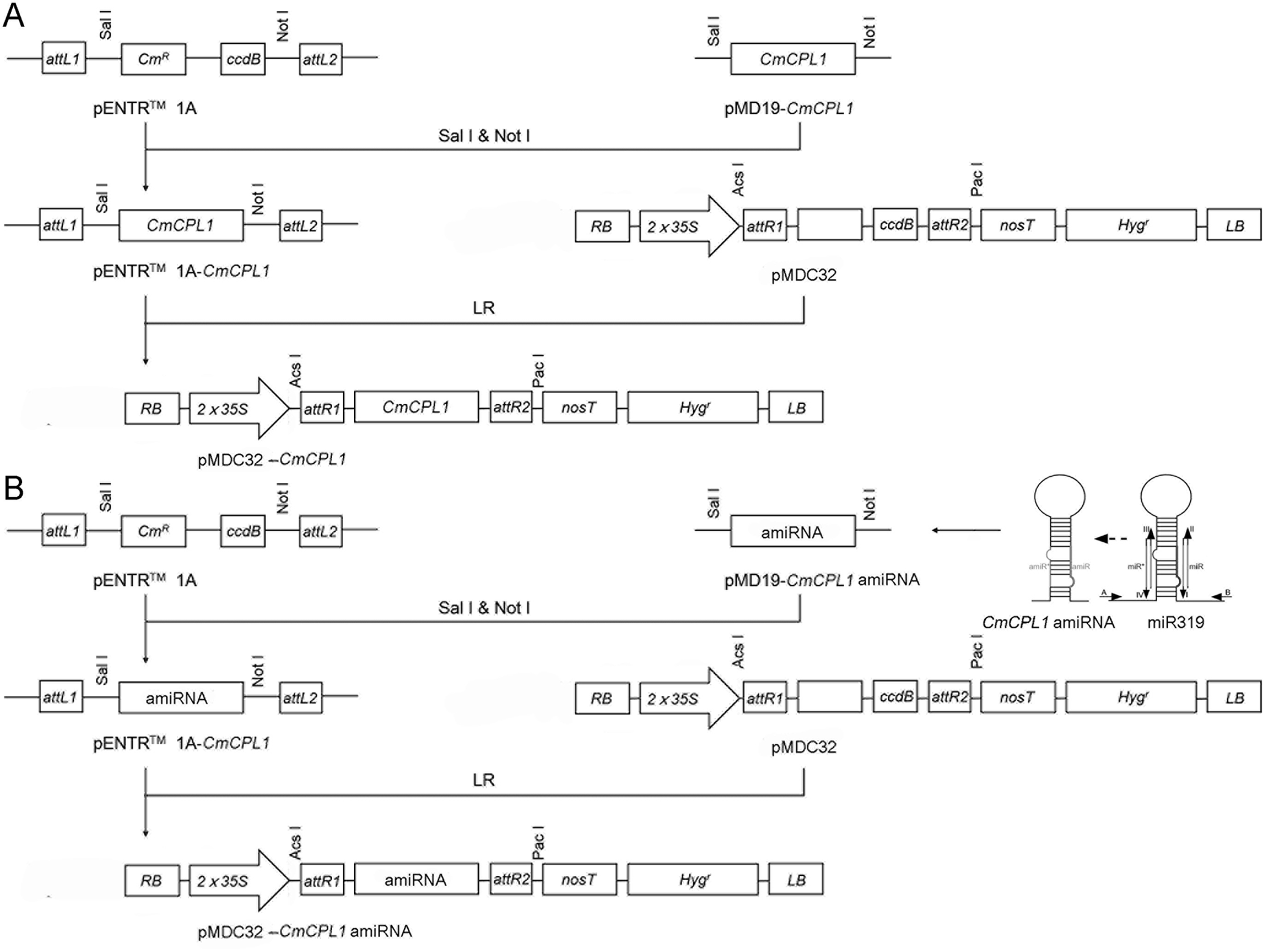

Supplement: Supplementary file 4 — Fiigure S3 [file 41438_2018_37_MOESM4_ESM.tif]

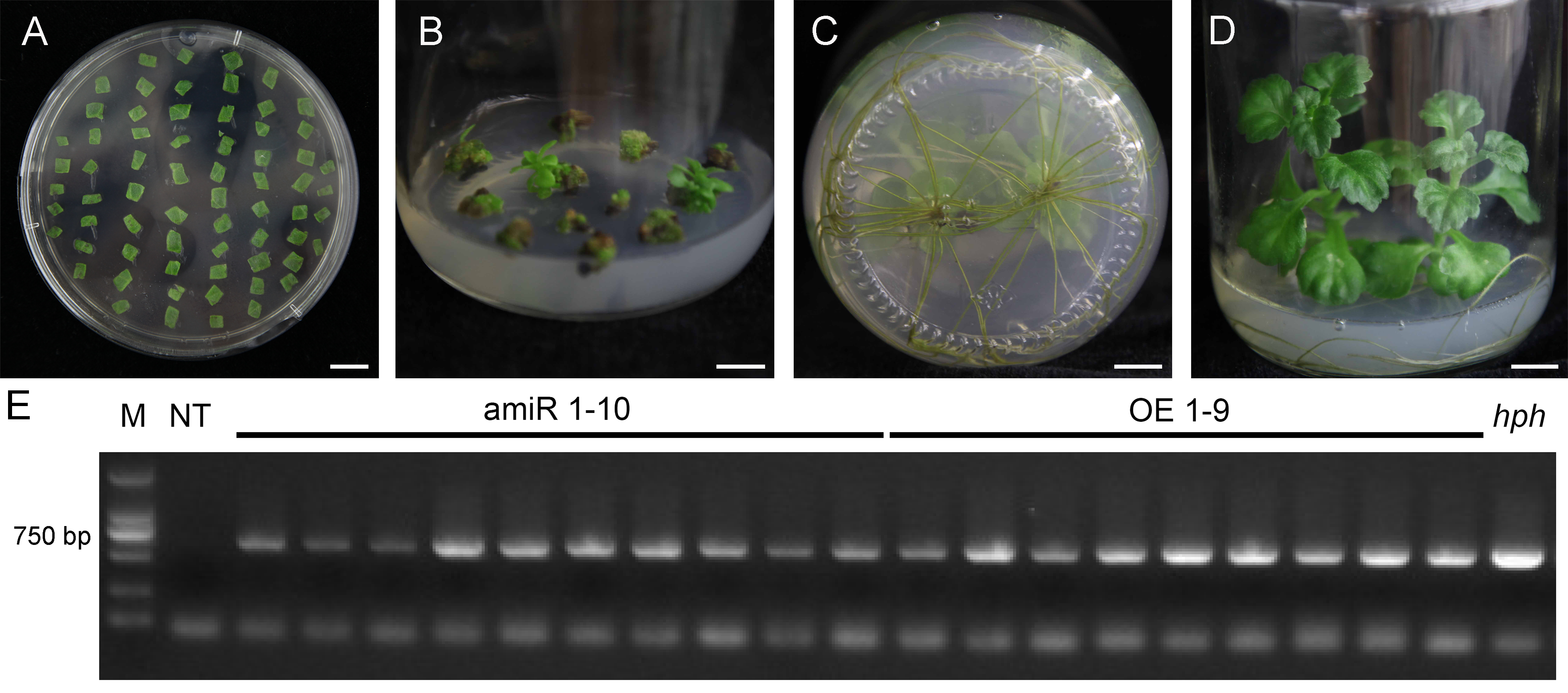

Supplement: Supplementary file 5 — Fiigure S4 [file 41438_2018_37_MOESM5_ESM.tif]

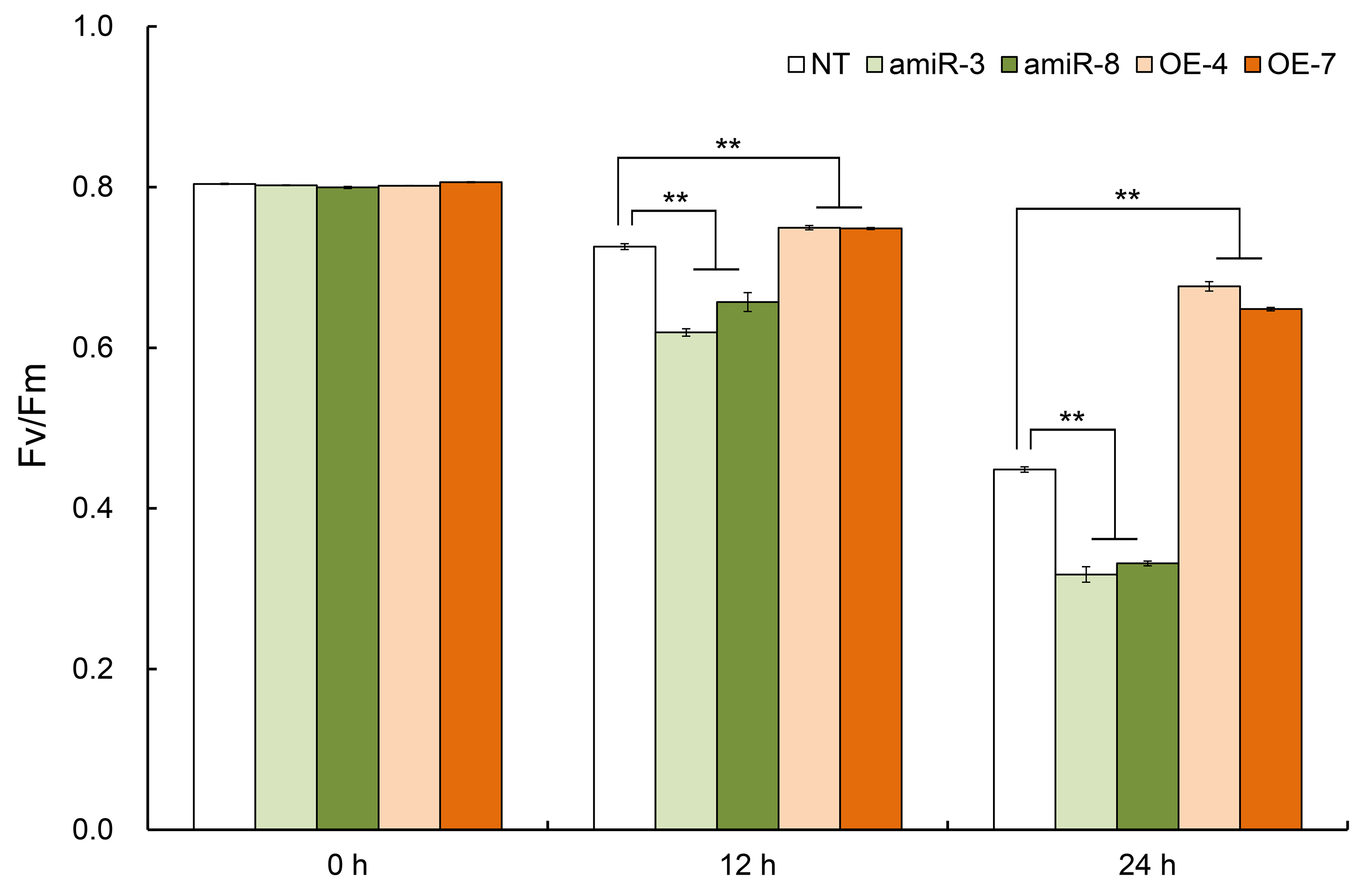

Supplement: Supplementary file 6 — Fiigure S5 [file 41438_2018_37_MOESM6_ESM.tif]

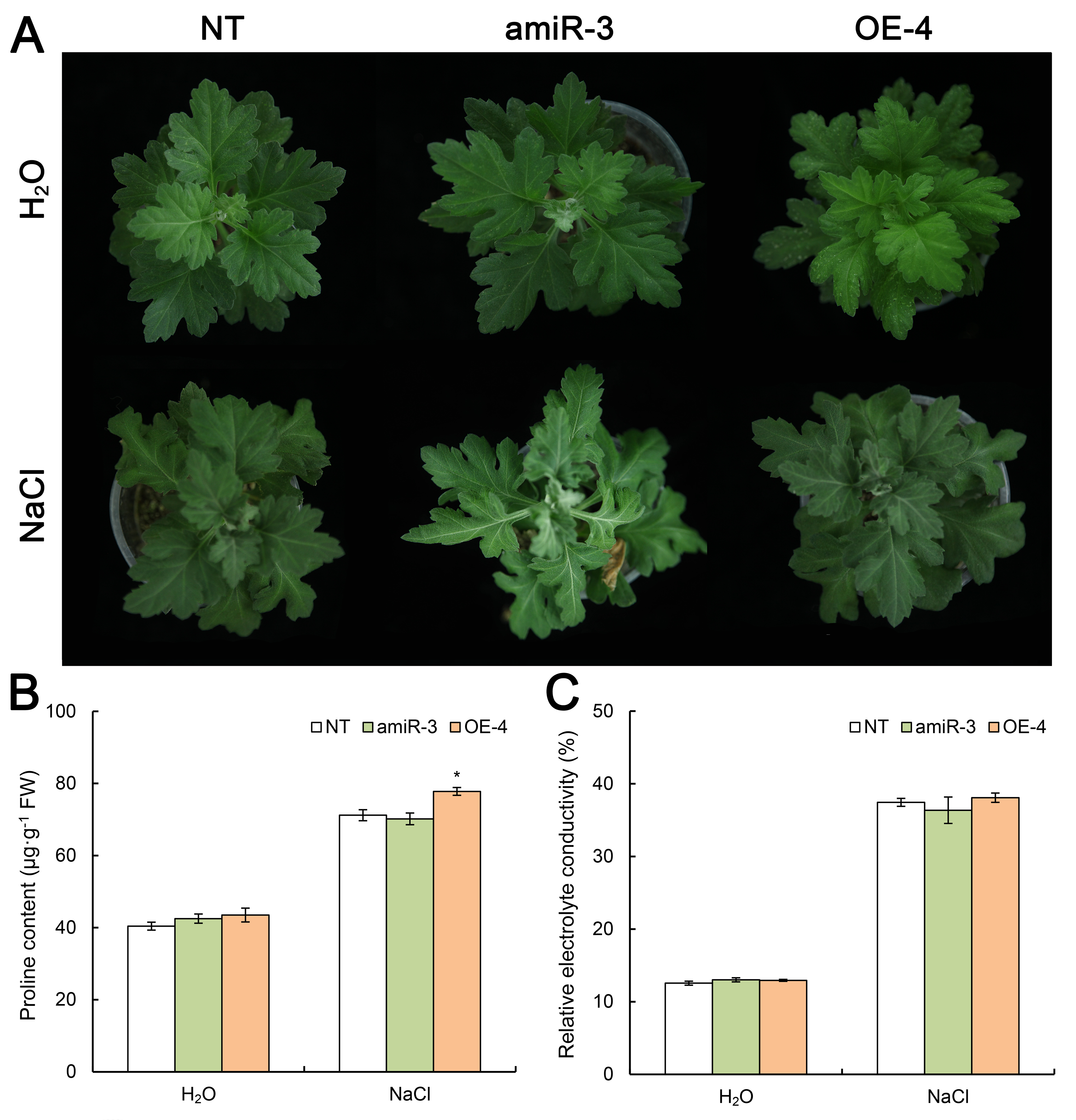

Supplement: Supplementary file 7 — Fiigure S6 [file 41438_2018_37_MOESM7_ESM.tif]
